# The Importance of Ectopic Mindedness: Scar Ectopic Pregnancy, a Diagnostic Dilemma

**DOI:** 10.7759/cureus.13089

**Published:** 2021-02-02

**Authors:** Vina Kumari, Himanshu Kumar, Mamta R Datta

**Affiliations:** 1 Obstetrics and Gynaecology, Tata Main Hospital, Jamshedpur, IND; 2 Anaesthesia, Tata Main Hospital, Jamshedpur, IND

**Keywords:** extratubal, scar ectopic, serum beta hcg, molar pregnancy, mri

## Abstract

There has been a consistent rise in ectopic pregnancies due to increase in maternal age, infertility treatment, cesarean sections, smoking among women, and use of contraceptives. With the extratubal ectopic pregnancies accounting for only 4% of the total ectopic pregnancies, scar pregnancies are even more of a rare entity with their incidence being less than 1%. We hereby present two cases of ectopic pregnancies, which though managed successfully presented a huge diagnostic challenge in the first case while the diagnosis was completely missed in the second case, hence, emphasizing the need for ectopic mindedness when dealing with early pregnancies.

## Introduction

Scar ectopic pregnancy, possibly due to implantation of blastocyst in the scarred or fibrosed uterine wall is a rare presentation with incidence being 1/800-1/2500 [1]. However, with the increase in rate of cesarean sections and in vitro fertilization (IVF) pregnancies, they are likely to present more frequently in the clinical practice. Hence, it is imperative to be ectopic minded and consider it in our differential diagnosis whenever presented with early pregnancy disorders or complications. If diagnosed early, there are options of conservative management or less invasive treatments. At the same time, they can present with life-threatening complications if the diagnosis is missed and may require extensive surgical interventions. This article compares two cases: first case in which a definitive diagnosis could not be reached despite being extensively investigated while in the second case the diagnosis was completely missed [2].

## Case presentation

Case 1

A 37-year-old para 2 live 2 presented with history of seven days overdue followed by painless vaginal bleeding for one month. With no other comorbidity, her periods had been regular with 3/28-30 days cycle with average blood flow. She had undergone two cesarean sections, last one six years earlier. She was hemodynamically stable and was advised ultrasound (henceforth ,USG) abdomen and pelvis and serum beta Hcg along with other routine blood and urine investigations. Her USG report was suggestive of a gestational trophoblastic disease like invasive mole which, however, did not corroborate with her low serum beta Hcg level (2104 mIU/mL).

**Figure 1:**
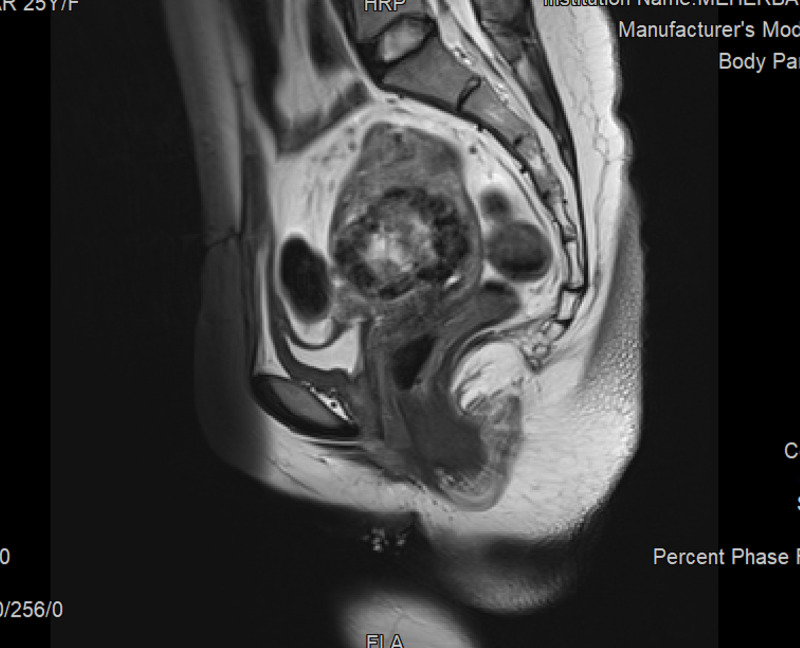
MRI report of Case 1 , showing scar ectopic pregnancy. The Type 2 scar ectopic is seen eroding the scar anteriorly which was seen as bulging scar intraoperatively. This was diagnosed as invasive mole by radiologists for the lack of definite gestational sac.

Hence, an MRI was done which reported a heterogenous mass in anterior myometrium in the lower segment again suggesting an invasive mole in view of thinned out myometrium (Figure 1).

Due to inconclusive diagnosis, the patient was followed up with serum beta Hcg level 48 hours later which showed a decreasing trend (Table 1).

**Table 1:** The serum beta Hcg value, baseline (28/2) and subsequently decreasing trend, not corroborating with the imaging diagnosis of molar pregnancy/invasive mole.

Date	28/2	2/3	16/3
Serum beta Hcg	2104 mIU/mL	1500 mIU/mL	302 mIU/mL

In view of diagnostic dilemma, the patient was taken up for hysteroscopy and tissue biopsy. Post-procedure, she had torrential vaginal bleed and needed a balloon tamponade. The biopsy reported few villi with hydropic changes, again suggesting molar pregnancy.

**Figure 2:**
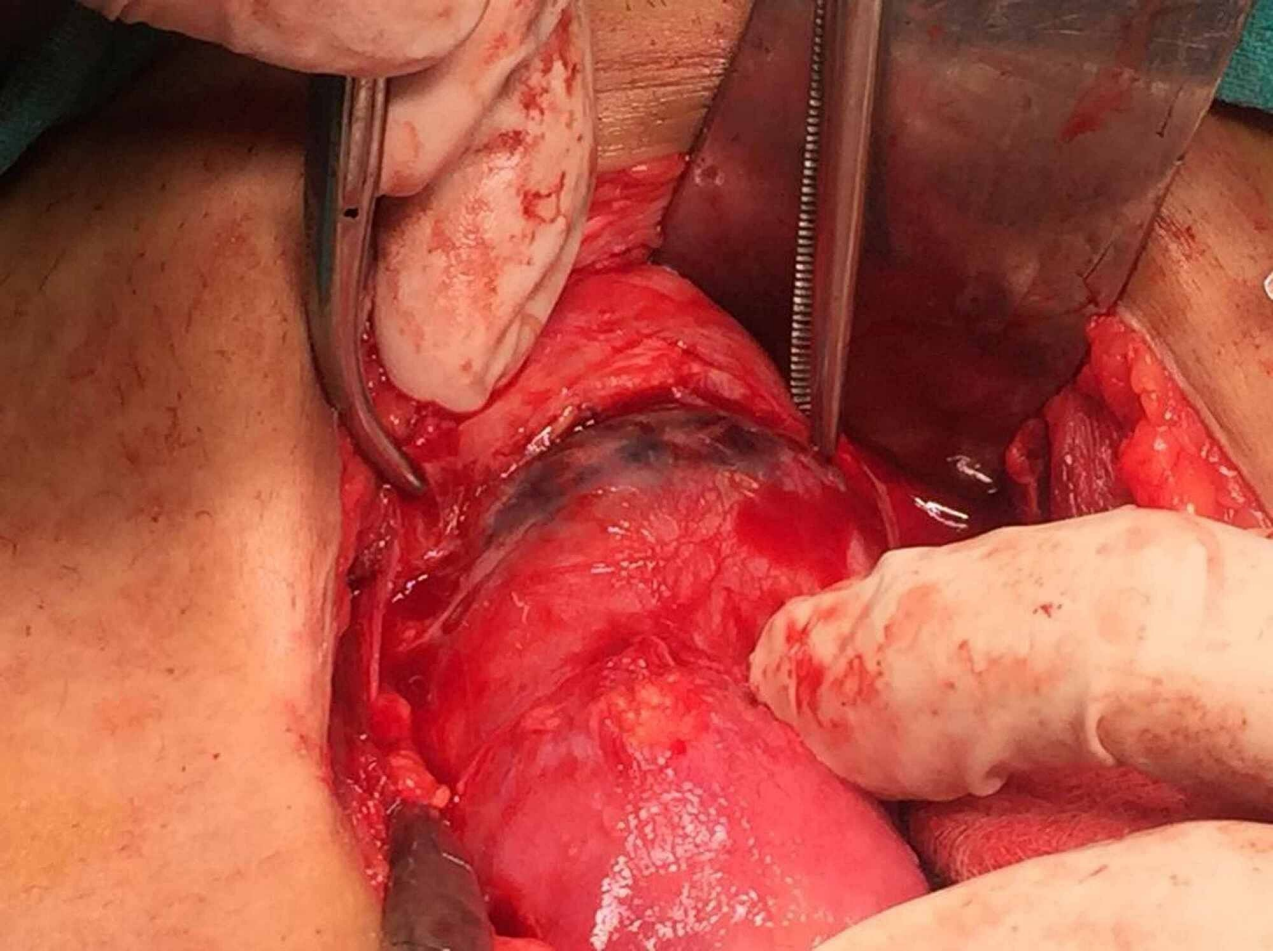
Intraoperative image of scar ectopic pregnancy with impending rupture and bulging scar seen in the lower segment.

However, as the bleeding did not stop and the patient did not intend further pregnancies, she consented for hysterectomy. A Type 2 scar ectopic with thinned out scar and impending rupture was noted during the procedure (Figure 2).

A fine dissection was carried out to separate the adherent bladder and omentum from the thinned out scar. The specimen was sent for histopathologic examination (HPE), which gave the final diagnosis of scar ectopic. The postoperative stay was uneventful.

Case 2

A 34-year-old para 1 live1 abortion 3 was admitted in labor room in hemorrhagic shock. She had undergone lower segment cesarean section (LSCS) four years back, followed by three surgical evacuations. She had taken medical termination of pregnancy (MTP) pills for termination of intrauterine pregnancy of 6w5d gestational age. A follow up USG showed a fetus of 10w5d indicating failed termination. Subsequently, she opted for surgical evacuation at a health center but unfortunately had profuse and intractable vaginal bleed leading to hemorrhagic shock. The USG revealed a bulky uterus with bulging lower segment full of clots and products of conception. Resuscitative measures were initiated and she was taken up for urgent laparotomy.

**Figure 3:**
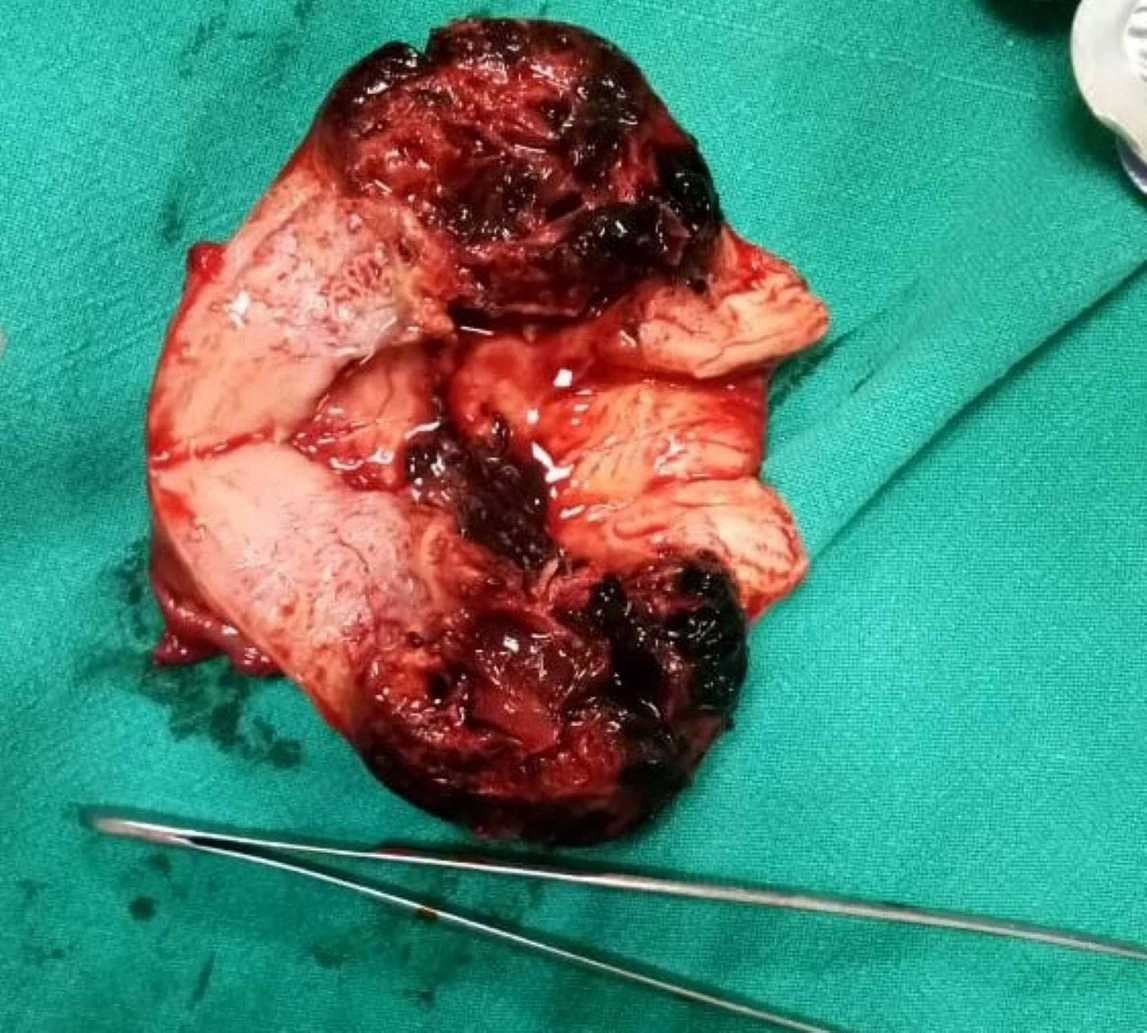
The cut section of the uterus shows typical features of scar ectopic: 1. Closed internal os. 2. Bleeding and clots at scar site. 3. Empty uterine cavity.

During the procedure, thinned out lower uterine segment was noted along with bladder and omentum adherent to the scar. As the bladder was separated, a ruptured Type 2 scar ectopic was noticed. A decision of hysterectomy was taken and the cut section revealed blood clots at the site of previous scar with rupture of serosal surface (Figure 3).

The HPE report of the specimen confirmed the diagnosis of scar ectopic. She was managed in the ICU postoperatively and discharged in a stable condition few days later.

## Discussion

Though the incidence of the scar ectopic is still low, 1/2000 [1], it is becoming an increasingly common presentation due to reasons mentioned earlier. Damage to the uterine endometrial or myometrial layer is the predisposing factor [2]. In a retrospective study of 291 cases by XianYi et al., the other predisposing or independent risk factors for cesarean scar pregnancies are : age more than 35 years, multigravida, cesarean sections performed in a rural setting, history of induced abortions after cesarean sections, and retroverted uterus [3]. Interestingly, increase in probability of scar pregnancy has no correlation to the number of earlier cesarean sections in the individual. The increased incidence may also be attributed to early and routine use of transvaginal USG. The most common presentation of scar pregnancy is painless vaginal bleed (55%) with a history of amenorrhoea. Few patients are asymptomatic (45%), while others may present with lower abdominal discomfort or pain (7%) requiring a high degree of suspicion for timely diagnosis.

Conventionally, scar pregnancy has been described as two types [4] in literature. Type I in which the progression is towards the uterine cavity (endogenic) and Type II in which there is deep implantation into scar defect and it progresses towards uterine serosal surface and has increased chances of rupture [5]. Similar one was witnessed in our case, where we could see the bulging scar ectopic during laparotomy.

Transvaginal/transabdominal USG with doppler along with a high degree of suspicion remains the key to diagnosis. The USG features typically include 1. Empty uterus along with empty cervical canal. 2. Development of gestational sac in the anterior part of lower uterine segment. 3. Absence/thinning of myometrium between bladder wall and gestational sac. 4. Increased doppler vascular flow in the area of cesarean delivery scar (high velocity and low impedance). 5. Negative sliding organs sign [6].

In our first case, though the USG findings were suggestive of scar pregnancy, a diagnosis of molar pregnancy was given by the radiologist probably due to very low incidence of scar pregnancy, hence, a low index of suspicion. However, as mentioned earlier, her serum beta Hcg levels did not match with the radiological diagnosis.

A sagittal T2 weighted MRI imaging remains the best modality to visualize a scar ectopic [7]. The characteristic features include 1. thinning of myometrium in the region of scar and 2. empty uterine cavity and cervical canal. In our case, though the MRI was suggestive of scar pregnancy, the absence of a gestational sac and may be, the nonconsideration of a possibility of scar pregnancy, due to its rarity, led the radiologist to give a diagnosis of invasive mole.

Any ectopic pregnancy has three forms of management: expectant, medical, and surgical [8]. Each case needs to be individualized based on patient history, need to preserve the uterus, and type of ectopic.

Expectant management is usually not preferred in scar ectopic pregnancy, and most of them (67%) require surgical interventions [8]. Methotrexate is commonly used for medical management. Need for prolonged follow up and high probability of surgical intervention including hysterectomy needs to be explained to the patient.

Type 2 scar ectopic pregnancy patients are a poor candidate for conservative treatment like Dilatation and Curettage, as there is a high chance of rupture and bleeding in these cases as in our second case. Literature shows that almost 60% of the conservatively managed cases had profuse vaginal bleed at some stage requiring surgical interventions [9]. Though, USG-guided suction and evacuation is an option, patient selection is of utmost importance and risk of torrential bleeding remains high. Laparoscopy, despite its advantages, may be technically challenging. Laparotomy followed by hysterotomy, freshening of scar, and resuturing is the mainstay of treatment. It also decreases the chances of recurrence. In both our cases the patients had completed their families and underwent hysterectomy.

Uterine artery chemoembolization, where available in conjunction with systemic or local methotrexate is a good option [10].

Follow up of patients, especially those managed with suction evacuation or hysterotomy is very important. If the patient conceives again, it is advisable to go for elective cesarean section and proper closure of the lower segment.

## Conclusions

Diagnosis and management of scar ectopic needs expertise and a multidisciplinary approach (radiologist for imaging, urologist for bladder adhesions to scar, especially in Type 2 scar ectopic, intensivist for postoperative management), along with ectopic mindedness and a high degree of suspicion. With the increasing cesarean rates in all the institutions, the incidence of scar pregnancy would definitely increase. A misdiagnosis or a delayed diagnosis might be life threatening. Thus, a detailed history, correct imaging technique, prompt and accurate diagnosis followed by individualized treatment and follow up is the mainstay of management of scar ectopic pregnancy.
